# Postoperative pregnancy in female achalasia patients: Report of three cases

**DOI:** 10.1016/j.ijscr.2021.01.076

**Published:** 2021-01-22

**Authors:** Yuto Kubo, Kiyokazu Nakajima, Kotaro Yamashita, Takuro Saito, Koji Tanaka, Tomoki Makino, Tsuyoshi Takahashi, Yukinori Kurokawa, Makoto Yamasaki, Hedetoshi Eguchi, Yuichiro Doki

**Affiliations:** a Department of Next Generation Endoscopic Intervention (Project ENGINE), Osaka University, Graduate School of Medicine, Osaka, Japan; b Department of Gastroenterological Surgery, Osaka University, Graduate School of Medicine, Osaka, Japan

**Keywords:** Achalasia, Surgery, Pregnancy

## Abstract

**Background:**

The aggravation of symptoms in female patients with esophageal achalasia has been sporadically reported to be associated with pregnancy. However, the relationship between symptoms recurrence and postoperative pregnancy after radical surgery remains unclear.

**Case presentation:**

There were 3 female achalasia patients who became pregnant after surgery between 1994 and 2018. Patient #1, #2 and #3 were 32, 27 and 25 years old, respectively. The main symptom was vomiting in #1, chest pain in #2, dysphagia in #3, the Eckardt score was 12, 9 and 7, respectively. The classification of achalasia was St grade Ⅱ in #1-2, St grade Ⅲ in #3. Laparoscopic Heller-Dor was underwent in all patients, there were no intraoperative and postoperative complications, and the symptoms improved in these patients. The Eckardt scores decreased to 2, 3 and 1, respectively. Each patient became pregnant 36, 24 and 46 months after surgery, and symptoms recurred during pregnancy in all patients. The Eckardt scores increased to 4, 5 and 4. These patients were followed without oral administration due to the risk of teratogenicity, and the pregnancies progressed smoothly. Healthy babies were delivered vaginally at 38–41 weeks. The symptoms in all patients were immediately improved after delivery, and there was no recurrence of symptoms thereafter.

**Conclusions:**

This case report showed that female patients who became pregnant after achalasia surgery had temporary symptom relapse during pregnancy. It was possible to continue pregnancy and deliver without treatment, and symptoms spontaneously improved immediately after delivery.

## Highlights

• The relationship between symptoms recurrence and postoperative pregnancy after surgery in achalasia patients remains unclear.
• This study retrospectively investigated 3 patients who became pregnant after surgery.
• All 3 patients who became pregnant after achalasia surgery had temporary symptom recurrence.
• However, these patients could delivery without any treatment, and their symptoms improved immediately after delivery.
• The pregnancy may have negative impact on temporary symptom and recurrence in achalasia patients after surgery.

## 1 Introduction

Esophageal achalasia is a neurogenic motility disorder of unknown etiology that induces impaired relaxation of the lower esophageal sphincter (LES) and loss of esophageal peristalsis [1]. The morbidity rate of esophageal achalasia is 1–3 patients per 100.000/year [[2], [3], [4]]. Patients have symptoms such as progressive dysphagia and reflux. Methods of treatment such as balloon dilation, laparoscopic Heller-Dor (LHD) and per-oral endoscopic myotomy (POEM) in achalasia have developed over the past few decades [5].

It has been suggested that since the peak incidence of achalasia is between 17 and 30 years [1,2,6], female achalasia patients can become pregnant during the illness, and symptoms may greatly affect pregnancy and childbirth. A previous study reported that 44–53% of patients with achalasia suffered from symptoms before pregnancy, and the general condition was significantly worse in early pregnancy and in untreated patients [7].

Few studies have shown that female patients with achalasia can get pregnant. Although surgery may be performed for achieving pregnancy and delivering safely in female patients with achalasia, the stability of symptoms after pregnancy has been unclear. In addition, the relationship between symptoms and pregnancy after achalasia surgery has been unknown. In this communication we describe clinical details of achalasia patients with postoperative pregnancy.

## 2 Presentation of case

There were 81 female patients who underwent LHD for achalasia between 1994 and 2018 in our hospital. 3 patients became pregnant after surgery, and all patients continued pregnancy and gave birth. The characteristics and treatment details before and after pregnancy are as follows. This case series has been reported in line with the PROCESS Guideline [8].

Patient #1, #2 and #3 were 32, 27 and 25 years old, respectively. No patients had any comorbidity, past medical, surgical, family, psychosocial and pharmacologic history. The symptoms were nausea and chest pain in #1, chest pain and dysphagia in #2, dysphagia in #3, the disease duration was 240, 36 and 65 months, respectively. The Eckardt score at diagnosis was 12, 9 and 7.

**Fig. 1:**
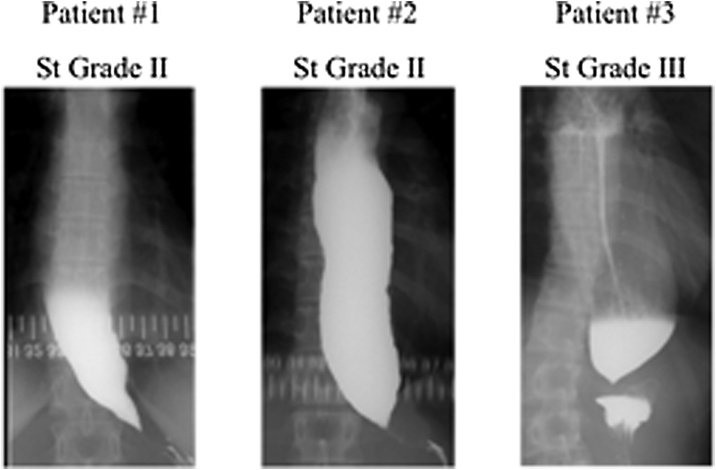
Achalasia classification by upper gastrointestinal imaging in all patients.

**Fig. 2:**
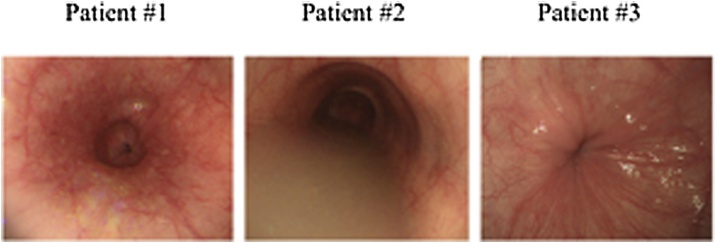
An upper gastrointestinal endoscopy in all patients.

The classification of achalasia based on upper gastrointestinal imaging [9,10] was St grade Ⅱ in #1 and #2, St grade Ⅲ in patient #3 (Fig. 1). An upper gastrointestinal endoscopy was advanced for all patients, #1 was showed dilation of the esophageal lumen and stenosis of the esophagogastric junction, #2 and #3 were indicated dilation and fluid storage in the esophagus and severe stenosis of the lower esophagus (Fig. 2). In the high-resolution manometry (HRM), #1 was showed that the integrated relaxation pressure (IRP) was 31.5 mmHg, the distal contractile integral (DCI) was 5819mmHg-s-cm, and the peristaltic wave disappearance was 100%, #2 was indicated the IRP and DCI were 24.7 mmHg and 5819mmHg-s-cm, respectively, and there was peristalsis failure of 100%. On the other hand, HRM could not be carried out for #3 because the catheter could not be inserted through the LES.

Patient #1 and #2 were diagnosed with type1 according to the Chicago classification [11]. #3 was diagnosed typical achalasia based on the esophagography findings and the insufficiency of the LES. Pre-treatment with oral administration was performed in only #2 (Ca blocker, 20 mg, tablet, twice a day). Because all patients had the severe symptoms and deterioration of the general condition, LHD was underwent for all patients [12]. Surgical time was 185–236 min. Estimated bleeding loss was 10 ml in 3 patients. There were no intraoperative and postoperative complications, and the symptoms improved in all patients. Regarding postoperative treatment, #2 toke Ca blocker (20 mg, tablet, twice a day) due to persistent chest pain. Postoperative Eckardt scores was improved to 2, 3 and 1, respectively (Table 1).

**Table 1:** Patient characteristics and treatment detail before pregnancy.

# Patient	#1	#2	#3
Age	32	27	25
BMI (preoperative)	18.2	18.3	18.0
ASA-PS	1	1	1
Comorbidities	None	None	None
Achalasia			
Main symptom	Nausea	Chest pain	Dysphagia
Disease duration (months)	240	36	65
Eckardt score	12	9	7
Classification for type	St	St	St
Classification for dilatation	Grade Ⅱ	Grade Ⅱ	Grade Ⅲ
HRM			
IRP (mmHg)	31.5	24.7	Not insertable
DCI (mmHg-s-cm)	5819	5653	Not insertable
Chicago classification	Type 1	Type 1	None
Preoperative treatment	None	Ca blocker	None
Surgery method	Laparoscopic Heller-Dor	Laparoscopic Heller-Dor (SILS)	Laparoscopic Heller-Dor (SILS)
Surgical time (min)	185	292	236
Estimated bleeding loss (ml)	10	10	10
Postoperative complications	None	None	None
Postoperative treatment	None	Ca blocker	None
Postoperative Eckardt score	2	3	1

BMI: body mass index, ASA-PS: American Society of Anesthesiologists- Physical Status, HRM: high resolution manometry, IRP: integrated relaxation pressure, DCI: distal contractile integral.

However, each patient became pregnant 36, 24 and 46 months after LHD, and symptoms recurred or exacerbated during pregnancy in all patients. The achalasia symptoms during pregnancy were nausea and chest pain in patient #1, chest pain in #2, dysphagia and chest pain in #3. The time of onset in achalasia symptoms after pregnancy was 14–19 weeks. The Eckardt scores after pregnancy increased to 4, 5 and 4, respectively. These patients were followed without medication due to the risk of teratogenicity although these symptoms continued until delivery. Pregnancy progressed smoothly, healthy babies were able to deliver vaginally at 38–41 weeks. In all patients, the symptoms were immediately improved after delivery, and there was no recurrence of symptoms thereafter (Table 2).

**Table 2:** Treatment detail after pregnancy.

# Patient	#1	#2	#3
Period until pregnancy after surgery (months)	36	24	46
Achalasia symptoms after pregnancy	Nausea, Chest pain	Chest pain	Dysphagia, Chest pain
Time of onset in achalasia symptoms after pregnancy (weeks)	14	15	19
Pregnancy period (weeks)	39	41	38
Eckardt score (during pregnancy)	4	5	4
Birth route	Transvaginal	Transvaginal	Transvaginal
Achalasia symptoms after delivery	None	None	None
Eckardt score (after pregnancy)	2	3	1

SILS: Single Incision Laparoscopic Surgery, PPI: proton pump inhibitor.

## 3 Discussion

Previous studies have shown the relationship between achalasia and pregnancy. However, since there was no previous study that reported postoperative pregnancy in female achalasia patients, the relationship between relapse of symptoms and pregnancy has been unclear. Our paper is the first to detailed case report that described the relationship between relapse of symptoms and pregnancy in achalasia patients underwent radical surgery.

Several studies reported that achalasia symptoms may be exacerbated with elevation of the diaphragm by the fetus during pregnancy [13,14]. Additionally, increasing the concentration of progesterone during pregnancy can lead to a decrease in smooth muscle motility and tension, gastric acid reflux into the esophagus can cause esophagitis [15]. In our series, pregnancy caused relapse of the same preoperative symptoms in all 3 patients, even though the symptoms improved after surgery. Though, the pregnancy progress was uneventful, and healthy babies were delivered transvaginally in each case. The symptoms improved immediately and did not recur after delivery. This case report showed that symptoms recurred or exacerbated at 3–5 months after pregnancy, suggesting that these symptoms were due to elevation of the diaphragm. We have considered that postoperative pregnancy may have no negative impact on entire course of achalasia, although the pregnancy may have negative impact on temporary symptom and recurrence in achalasia patients after surgery.

The surgical procedure for esophageal achalasia mainly includes LHD and POEM [16]. The percentage of POEM procedures performed has gradually increased due to the development of endoscopic technology in recent years [17,18]. All 3 patients who underwent LHD did not have postoperative complications and had stable symptoms before becoming pregnant. The gastroesophageal reflux (GER) increases due to elevation of intra-abdominal pressure and the diaphragm by the fetus during pregnancy, LHD combined with anti-reflux therapy may be a better treatment for female patients who wish to become pregnant, considering GER during pregnancy.

These patients were aware of achalasia-like symptoms during pregnancy, and weight loss and eating disorders were relatively mild. Therefore, we followed the patients conservatively due to the risk of teratogenicity; their pregnancies continued, and normal vaginal deliveries were achieved in all patients. We suggest that watchful follow-up alone may be possible for pregnant patients with recurrent achalasia symptoms after surgery. However, it might be necessary to validate treatment policies for patients who have severe achalasia symptoms during pregnancy.

There are several limitations in this report. First, it is difficult to diagnose whether achalasia symptoms have recurred or worsened during pregnancy because gastrointestinal symptoms in pregnant patients are often due to hyperemesis gravidarum [19,20]. However, we consider that pregnancy causes the achalasia symptoms to recur or exacerbate, because symptoms peculiar to achalasia such as chest pain recurred or got worse after pregnancy in all patients were similar to preoperative symptom. Second, in our series, achalasia symptoms during pregnancy were diagnosed only subjectively, without objective evaluation. However, HRM and upper gastrointestinal imaging and endoscopy may have negative impact on pregnancy and childbirth. Therefore, we did not evaluate these patients by objective evaluation.

## 4 Conclusion

Female patients who became pregnant after achalasia surgery had temporary symptom recurrence. However, these patients were able to continue pregnancy and deliver without any treatment, and their symptoms improved immediately after delivery.

## Declaration of Competing Interest

The authors report no declarations of interest.

## Sources of funding

None.

## Ethical approval

None.

## Consent

Consent has been obtained.

## Author contribution

Y.K. and K.N. conceptualized the project, designed and per-formed the experiments, interpreted the results, and co-wrote the manuscript. Y.K. supervised the experimental design and interpreted the results. N.K. and T.T. performed the surgeries and prepared the culture samples. Y.K., K.N., T.T., H.E., and Y.D. analysed data or participated in discussions of the results.

## Registration of research studies

researchregistry6450 available at: https://www.researchregistry.com/browse-the-registry#home/registrationdetails/5ffecd670e3589001b81f3a7/.

## Guarantor

Kiyokazu Nakajima.

## Provenance and peer review

Not commissioned, externally peer-reviewed.

## CRediT authorship contribution statement

**Yuto Kubo:** Writing - review & editing, Validation. **Kiyokazu Nakajima:** Conceptualization, Supervision, Project administration. **Kotaro Yamashita:** Validation. **Takuro Saito:** Formal analysis, Validation. **Koji Tanaka:** Formal analysis, Validation. **Tomoki Makino:** Formal analysis, Validation. **Tsuyoshi Takahashi:** Formal analysis, Validation. **Yukinori Kurokawa:** Formal analysis, Validation. **Makoto Yamasaki:** Formal analysis, Validation. **Hedetoshi Eguchi:** Formal analysis, Validation. **Yuichiro Doki:** Formal analysis, Validation.
